# A Mobile Sperm Analyzer with User-Friendly Microfluidic Chips for Rapid On-Farm Semen Evaluation

**DOI:** 10.3390/bios15060394

**Published:** 2025-06-18

**Authors:** Shu-Sheng Lin, Chang-Yu Chen, Cheng-Ming Lin, Tsun-Chao Chiang, Yu-Siang Tang, Chang-Ching Yeh, Wei-Fan Hsu, Andrew M. Wo

**Affiliations:** 1 Institute of Applied Mechanics, National Taiwan University, Taipei 106319, Taiwan; d01543007@ntu.edu.tw (S.-S.L.); d08543010@ntu.edu.tw (W.-F.H.); 2 Aidmics Biotechnology, Taipei 10647, Taiwan; cychen@aidmics.com (C.-Y.C.); cmlin@aidmics.com (C.-M.L.); chiang@aidmics.com (T.-C.C.); kt@aidmics.com (Y.-S.T.); yp@aidmics.com (C.-C.Y.)

**Keywords:** portable sperm analysis system, boar semen analysis, on-farm point-of-care testing, microfluidic chip, image processing, user variability assessment, ROC and regression analysis

## Abstract

This study presents a mobile-based sperm analysis system featuring a user-friendly, droplet-loaded microfluidic chip that enables non-specialist users to perform the rapid and accurate quantitative evaluation of boar semen directly on the farm. The iSperm system integrates a tablet, optical module, heater, and real-time image analysis app to deliver automated measurements of sperm concentration, motility, and progressive motility in under one minute. Precision and user variability tests demonstrated high concordance with CASA and the hemocytometer, with minimal differences between trained and untrained users. A method comparison using 77 farm-collected samples confirmed agreement through Passing–Bablok regression and Bland–Altman analysis. ROC curve analyses further validated diagnostic accuracy for all parameters, with AUC values exceeding 0.95. The iSperm platform offers a reliable, user-friendly, and field-deployable solution for on-site semen quality assessment, improving decision-making in swine artificial insemination.

## 1. Introduction

Semen evaluation is essential for assessing male fertility and optimizing artificial insemination (AI) in livestock breeding. Currently, over 93% of sows in pig-producing countries are impregnated using AI, and more than 99% of these procedures rely on extended liquid semen, stored for up to five days [1,2,3]. The collected semen must be quickly processed to prevent exposure to contaminants, temperature fluctuations, or prolonged oxygen exposure, all of which can negatively impact sperm viability. However, improper semen dilution, preservation, and transportation can lead to quality deterioration, making accurate semen evaluation prior to AI indispensable [4,5].

Traditional methods for assessing sperm concentration, motility and progressive motility involve manual techniques, such as photometric concentration measurements, hemocytometer-based counting, and microscopic evaluations of motility. These approaches are often subjective, time-consuming, and prone to inconsistency, particularly when performed by untrained technicians [6,7,8]. In response, computer-aided sperm analysis (CASA) systems were developed to provide objective and precise measurements of sperm characteristics, including concentration, motility, morphology, and kinetic parameters [9,10,11]. Despite their improved accuracy, CASA systems are expensive, require specialized training, and are predominantly limited to laboratory settings [12,13,14].

A critical aspect of semen analysis with CASA involves accurate cell counting, particularly in dynamic samples, where chamber constraints can impact precision. Capillary-loaded slides, such as hemocytometers or Countess cell counting chambers, are commonly used, but these methods struggle with dynamic cell measurements due to chamber height constraints [15]. Capillary flow through a 20-μm chamber follows Poiseuille flow, which induces the Segre–Silberberg (SS) effect, causing uneven cell distribution within the chamber [16,17,18,19,20]. While current methods, such as capillary-loaded slides, are well established and effective when operated by highly trained personnel, they can be sensitive to sample variability and require strict adherence to standardized procedures to ensure reproducibility. For less experienced operators, even small deviations from the protocol may result in significant variability, limiting their practicality in field applications [12,13,14,21].

Most current systems and recent smartphone-based semen analysis platforms continue to rely on capillary-loaded slides, including those by Zheng et al. (2023), Suárez-Trujillo et al. (2022), and Kanakasabapathy et al. (2017), all of which lack temperature control, potentially limiting their suitability for field conditions [6,22,23,24]. This is particularly critical for motility assessment prior to artificial insemination, as sperm activity is highly temperature-sensitive. Meanwhile, advances in mobile-based bioanalytical devices have led to lightweight, cost-effective, and increasingly automated tools for on-site biomedical applications [25,26]. These platforms often integrate compact optical modules with microfluidic components, enabling reduced sample volumes and broader accessibility outside laboratory settings. As demand grows for point-of-care diagnostics in both clinical and agricultural domains, such innovations are transforming fields like reproductive biology, particularly by supporting livestock fertility assessment and decentralized semen quality monitoring [27,28,29].

Building on these advances and addressing the limitations of existing semen analyzers, we developed iSperm, a portable sperm analyzer designed for on-farm evaluation in boar AI programs. The system integrates a tablet-mounted custom optical module, droplet-loaded microchips, and a built-in heating module to ensure precise sample handling, temperature stabilization, and reproducible loading. The sealed chip minimizes user-induced variability, and a simple dropper-based loading mechanism enhances usability for non-specialist operators. A built-in app automatically analyzes four microscopic fields, producing results, including concentration, motility, and progressive motility, within one minute.

To evaluate iSperm’s performance, we first conducted precision and user variability assessments using semen samples from a boar at a commercial AI center. Results showed a high degree of correlation between iSperm, CASA, and hemocytometer methods in measuring sperm concentration. In the user variability study, the coefficient of variation for sperm concentration measurements was significantly lower with iSperm than with CASA when the sample loading was performed by untrained users. Moreover, similarly low variation was observed across both trained and untrained analyzing with iSperm, underscoring the system’s robustness and operator independence. For further validation, 77 boar semen samples collected under field conditions were analyzed. Passing–Bablok regression demonstrated high agreement between iSperm and CASA, while Bland–Altman analysis confirmed minimal systematic bias [30,31]. Receiver operating characteristic (ROC) analyses further confirmed iSperm’s diagnostic accuracy, with all AUC values exceeding 0.95 for concentration, total motility, and progressive motility [1,32,33]. These findings support the potential of iSperm as a robust, user-friendly tool for routine semen evaluation in boar AI programs.

## 2. Materials and Methods

### 2.1. Design of the iSperm Analyzer

**Figure 1:**
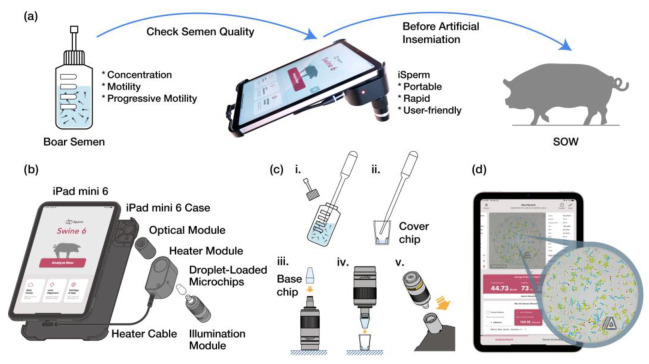
The process flow for semen analysis using iSperm prior to sow artificial insemination. (**a**) iSperm system for evaluating sperm concentration, motility, and progressive motility in boar semen, ensuring semen quality at sow farms before AI. (**b**) Schematic diagram of the iSperm analyzer, including an exploded assembly drawing showing how the components of iSperm fit together. (**c**) Installation and operation of the droplet-loaded microchips within the iSperm system. (**i**) Semen is gently mixed to ensure uniformity before sampling. (**ii**) A dropper transfers ~50 µL of sample into the cover chip. (**iii**) The base chip is mounted onto the illumination module. (**iv**) The base and cover chips are assembled to encapsulate the sample. (**v**) The completed microchip module is inserted into the optical unit for analysis. (**d**) Software interface displaying analysis results and sperm tracks visualized in four distinct colors.

The objective of this study was to present a mobile fertility analyzer that integrates optical components and microfluidic techniques to enable accurate on-site measurement of sperm concentration, motility, and progressive motility. This system is specifically designed for use in artificial insemination (AI) programs, particularly for evaluating boar semen samples loaded into disposable droplet-loaded microchips. The iPad mini 6 was selected as the mobile platform for the iSperm system due to its portability, processing power, and moderate screen size, which enhances user interaction and facilitates easier sample analysis. As shown in Figure 1a, the iSperm system evaluates boar semen quality for AI at sow farms, measuring sperm concentration, motility, and progressive motility.

As illustrated in Figure 1b, the iSperm analyzer comprises several key components: a custom-designed optical module mounted onto an iPad mini 6 case, a set of droplet-loaded microchips, and integrated software for image capture and analysis. The optical module, which attaches directly to the tablet, includes an optical lens that aligns with the tablet’s camera lens, producing an enlarged image of sperm cells. The illumination module features a light pipe with a pinhole opening and an LED connected to a power source consisting of three LR44 batteries (Panasonic Corp., Osaka, Japan), ensuring adequate sample illumination (Figure S1). Additionally, a heating ring is integrated into the optical module to maintain the semen sample at a stable temperature of 37.5 °C during analysis, with power supplied via a cable connected to the iPad mini 6.

Sample preparation involves using a dropper to load approximately 50 µL of semen into the cover chip. The base chip is then securely inserted into the cover chip, encapsulating the sample before it is mounted onto the optical module (Figure 1c). An illumination module is used to illuminate the sample during image capture. The iSperm software application (Version 6.4.0), running on the iPad mini 6 (Apple, Cupertino, CA, USA) with iOS 15.5, performs a four-view analysis of the captured images, delivering results within one minute (Figure 1d).

### 2.2. Optical Module

**Figure 2:**
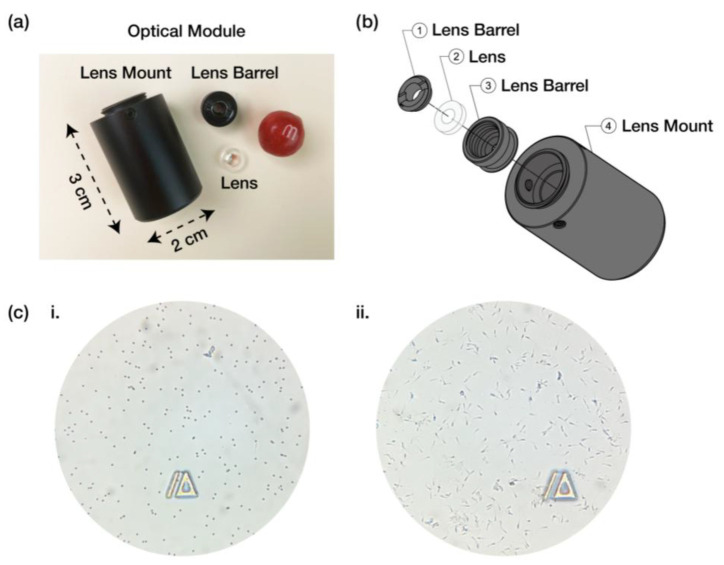
Optical module of the iSperm system. (**a**) The assembled optical module (3 cm × 2 cm), designed for compact and portable use with a tablet camera. (**b**) Components of the optical module shown in an exploded view. (**c**) Images captured using the iSperm optical module: (**i**) 5 µm latex beads and (**ii**) boar spermatozoa.

The optical module of the iSperm analyzer was designed to align with the camera of an iPad mini 6, facilitating the magnification and capture of sperm images for analysis. As shown in Figure 2a,b, the optical module consists of several key components: the lens barrel, the lens, and the lens mount.

The optical module’s compact design (3 cm height and 2 cm diameter as shown in Figure 2a ensures it is lightweight and portable, essential for on-farm use. This design features an integrated cavity that accommodates the droplet-loaded microchips securely while housing the light source. This arrangement not only minimizes the overall footprint but also enhances system stability and functionality, achieving a lightweight, portable, and user-friendly solution suitable for field applications. The lens barrel and mount are fabricated from high-quality aluminum alloy for strength and lightweight properties, while the lens is injection-molded from durable poly methyl methacrylate (PMMA) to ensure clarity.

The lens barrel (Figure 2b, components ① and ②) holds the lens in place and ensures precise alignment with the camera lens of the iPad mini 6. The lens itself (Figure 2b, component ②) is custom-designed to provide the necessary magnification for sperm analysis, allowing for detailed imaging of individual sperm cells. The lens mount (Figure 2b, component ➃) securely attaches the optical module to the iPad case, maintaining stability during sample analysis.

To evaluate the optical module’s performance, 5-µm latex beads (Accu-Beads^®^, Hamilton Thorne, Beverly, MA, USA), chosen for their size similarity to boar sperm heads, were used to assess imaging consistency (Figure 2(ci)). The iSperm optical module demonstrated clear visualization of the 5 µm latex beads, confirming its ability to capture fine details. Additionally, boar sperm images (Figure 2(cii)) were captured, illustrating the module’s capability to provide clear and detailed images essential for downstream semen analysis. Figure S2 showcases sperm images from different species, including canine, avian, and murine sperm.

### 2.3. Droplet-Loaded Microchips

**Figure 3:**
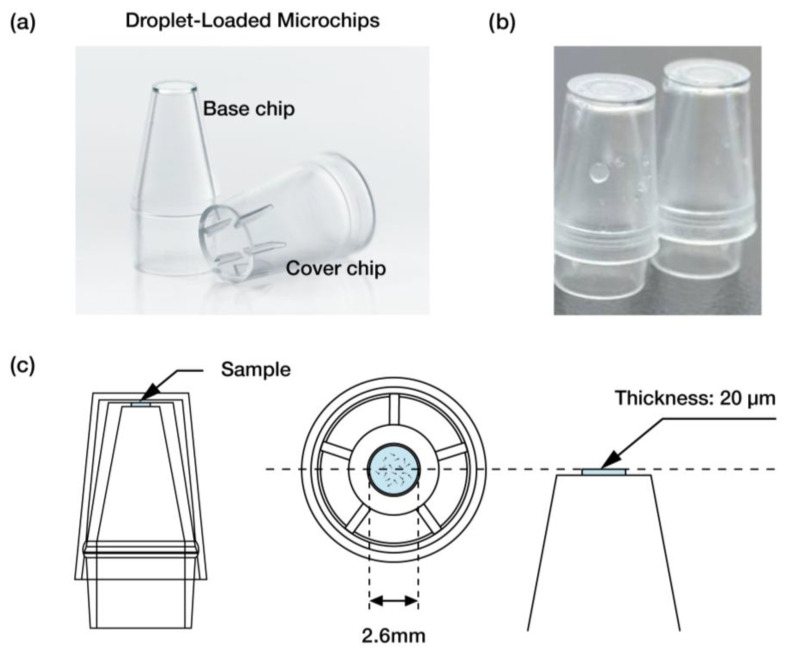
Droplet-Loaded Microchips used in the iSperm. (**a**) The base and cover chips, injection-molded from polycarbonate (PC), form the droplet-loaded microchip. (**b**) Assembled microchips ready for insertion into the iSperm optical module. (**c**) Schematic of the sample chamber with a 20 µm thickness and 2.6 mm diameter, designed for precise sperm analysis.

The droplet-loaded microchips used in the iSperm analyzer were specifically designed to facilitate precise and user-friendly sample handling for on-site semen analysis. These single-use microchips consist of two primary components: the base chip and the cover chip, as illustrated in Figure 3a. Both components are injection-molded from polycarbonate (PC), a material known for its optical clarity, ensuring accurate optical measurements for each use in field conditions. Once the semen is loaded, the assembled droplet-loaded microchips are inserted into the iSperm optical module for analysis (Figure 3b).

To prepare the sample, approximately 50 µL of boar semen is deposited into the sample chamber in the cover chip using a dropper. The base chip is then securely inserted into the cover chip, sealing the sample within a thin layer. The sample chamber, with a thickness of 20 µm and a diameter of 2.6 mm, is designed to facilitate optical analysis by promoting uniform layering of the sperm cells for imaging (Figure 3c). This droplet-loaded design provides consistent sample handling, which is critical for maintaining the integrity of on-site evaluations for both trained and untrained users.

### 2.4. Heater

**Figure 4:**
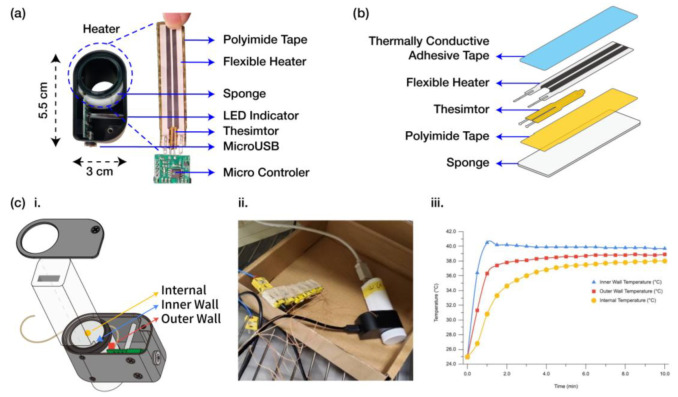
Heater module for the iSperm. (**a**) Exploded view showing the construction of the heater module, secured with key components and housed in a compact 5.5 cm × 3 cm enclosure. (**b**) Layered assembly of the heater system, showing the thermally conductive adhesive tape, flexible heater, thermistor, polyimide tape, and sponge used for insulation. (**ci**) Exploded schematic showing the placement of the heater module and temperature sensors in relation to the test device. (**cii**) Photograph of the experimental setup, demonstrating the heater system configuration during testing. (**cii**) Temperature profiles demonstrating the system’s ability to maintain the target temperature of 37.5 °C within approximately 5 min.

A flexible heater module was designed as a separate module to provide precise temperature control for the iSperm analyzer during semen analysis. The heater module consists of a polyamide-taped flexible heater and a thermistor assembly, secured using thermally conductive adhesive tape. A sponge layer, as shown in Figure 4a,b, was incorporated to minimize heat dissipation and improve thermal efficiency. The entire heating assembly is housed in a compact enclosure (5.5 cm × 3 cm) constructed from machined aluminum, ensuring lightweight and portable performance for on-farm use.

The heater’s output is regulated by a microcontroller using Pulse Width Modulation (PWM), which adjusts power delivery based on real-time temperature feedback from the thermistor. This setup allows the system to maintain the semen sample at an approximate temperature of 37.5 °C, which is critical for accurate sperm motility and progressive motility assessments. An LED indicator is included in the system to signal when the target temperature is reached and stable.

Temperature monitoring was conducted at three key points: the internal sample area, the inner surface of the heater module, and the outer surface of the heater module, as illustrated in Figure 4(ci). The heater module’s design allows it to reach the target temperature of 37.5 °C within approximately 5 min and maintain a steady state, as shown by the temperature profiles in Figure 4(ciii). The exploded view of the heater assembly and the placement of the sensors are detailed in Figure 4(cii), and a photograph of the experimental setup is provided in Figure 4(ciii). The integration of this precise temperature control mechanism underscores the system’s suitability for consistent on-site semen quality evaluations.

### 2.5. Software Application

**Figure 5:**
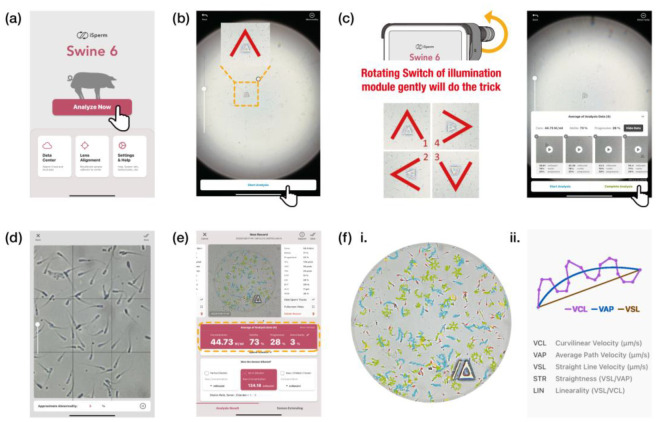
Software application interface and analysis process of the iSperm. (**a**) The main screen of the iSperm software on an iPad mini 6, providing easy access to key functions such as “Analyze Now.” (**b**) User interface guiding the alignment of the semen sample. (**c**) Visual feedback during analysis, guiding users to adjust the sample position across multiple fields of view. (**d**) Representative view of multiple fields displayed in a nine-grid layout to assist visual inspection. (**e**) Analysis results displayed on the iSperm interface, including sperm concentration, motility, progressive motility. (**f**(**i**)) Example of sperm tracking output generated by the iSperm system, highlighting different trajectories; (red: static sperm; green: motile sperm; blue: progressive sperm; yellow: late-track sperm). (**f**(**ii**)) Schematic diagram of velocity parameters.

The iSperm analyzer operates via a custom-designed software application on an iPad mini 6, enabling on-site semen analysis. The software provides an intuitive user interface that guides users through the entire process, from sample alignment to data analysis. Figure 5 demonstrates how the iSperm system processes sperm video footage to provide comprehensive sperm quality information to the user directly on the tablet screen. As shown in Figure 5a, the main screen offers direct access to functions such as “Analyze Now” for rapid operation.

During analysis, the software assists in aligning the sample for imaging, as illustrated in Figure 5b, a critical step for consistent and accurate results. The software performs a four-view analysis to increase sampling frames, calculating sperm concentration, motility, and progressive motility (Figure 5c). The software captures and analyzes sperm videos at 60 fps with a 280 × 280 × 3.14 µm^2^ field of view, delivering results within one minute. Additionally, a nine-grid view is provided to assist users in visually inspecting sperm images from multiple fields, as shown in Figure 5d.

The sperm motion parameters, including velocity average path (VAP, μm/s), velocity straight line (VSL, μm/s), velocity curvilinear (VCL, μm/s), straightness (STR, %), and linearity (LIN, %), were calculated using 60 fps image capture (Figure 5(fii)). VAP represents the average velocity along the smoothed sperm path, VSL is the straight-line velocity from the start to the end of the track, VCL is the actual point-to-point velocity along the track, STR reflects how closely the path follows a straight line, and LIN indicates the degree to which the movement is linear relative to the curved path. Straightness (STR) is calculated as the ratio of the straight-line distance between the initial and final positions to the total path length traveled. In terms of integration over time, it can be expressed as:

$$STR=\frac{VSL}{VAP}=\frac{\left|r(T)-r(0)\right|}{\int_{0}^{T}\left|\frac{{dr}_{avg}(t)}{dt}\right|dt}$$

where r(T) and r(0) are the final and initial positions, and the denominator represents the total distance traveled along the path over the time period T., $r_{avg}(t)$: this represents the average spatial position of the sperm head at time t., T: this is the total duration of the sperm’s movement., $\frac{{dr}_{avg}(t)}{dt}$: This denotes the instantaneous velocity of the sperm head at time t.

**Figure 6:**
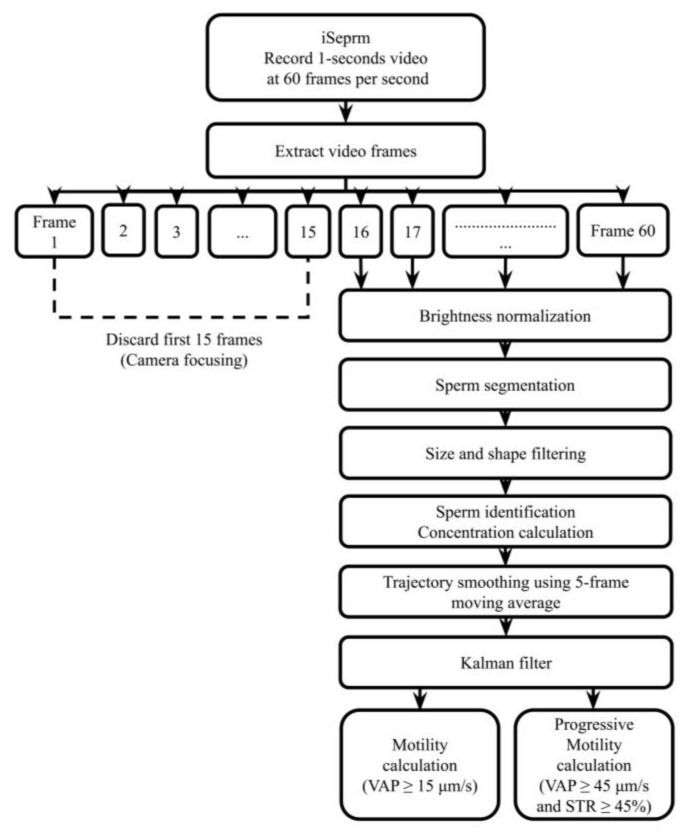
Image processing pipeline used in the iSperm system.

A schematic flowchart of the processing pipeline is shown in Figure 6. As illustrated in the software workflow diagram, iSperm records a 1s video at 60 frames per second. The first 15 frames were discarded to allow the iPad mini’s built-in autofocus to stabilize prior to concentration, motility, and progressive motility analysis. From the remaining frames, sperm are segmented and filtered based on size and shape. Sperm positions are then tracked frame-by-frame. To calculate motility parameters such as average path velocity (VAP) and progressive motility, we apply a 5-frame moving average to smooth each sperm’s trajectory. This smoothed path is then used to compute motion characteristics. To further refine tracking accuracy and reduce noise, we apply a Kalman filter, a recursive estimation algorithm commonly used in motion prediction tasks, which helps maintain robust tracking of each sperm across frames, particularly in low-contrast or overlapping regions. This pipeline allows for consistent detection and quantification of motility metrics in real time through the integrated mobile application.

Based on Hamilton Thorne Inc.’s recommended settings, adjustments were made to the VAP and STR cutoff values for progressive motility. Images were captured at 60 frames per second, with a total of 45 frames recorded per sample. The cutoff for progressive sperm was set at VAP ≥ 45 μm/s and STR ≥ 45%, while sperm with VAP ≥ 15 μm/s were classified as motile, and those with VAP < 15 μm/s were considered static. Upon completion, results are displayed in a clear, color-coded format, with sperm tracks and key parameters highlighted (Figure 5e). The color-coding scheme differentiates between various sperm states: static sperm are marked in red, motile sperm in green, progressively motile sperm in blue, and late-track sperm in yellow (Figure 6).

### 2.6. Precision and User Variability Assessment 

Semen samples were collected from a boar at a commercial artificial insemination (AI) center to evaluate the precision and user variability of the iSperm analyzer in measuring sperm concentration. Each fresh ejaculate was diluted 1:1 with a commercial semen extender (Semengra Semen Extender, China Chemical & Pharmaceutical Co. (Taipei, Taiwan)) and transported at 16 °C to a laboratory for analysis. The samples were divided into two subgroups: one to evaluate the limits of sperm concentration across different instruments and the other to assess user variability. Five trained users with prior experience operating CASA and iSperm systems, and five untrained users (ages 25–45) with no scientific background, participated in the study. Untrained users were provided with instructional videos demonstrating the iSperm operation procedure (Movies S1 and S2), as well as a separate video detailing CASA slide loading, prior to testing.

#### 2.6.1. Precision Comparison

To ensure accurate assessment, semen samples were initially measured using a hemocytometer to verify their concentration. After confirmation, the samples were further diluted into nine concentration ranges (10 to 75 million sperm/mL) based on hemocytometer measurements. These samples were then analyzed using both the iSperm analyzer and the CASA system. For each concentration range, measurements were repeated three times for both systems. The precision and agreement across different methodologies were evaluated and visualized using linear plots to illustrate trends and variability in sperm concentration measurements.

#### 2.6.2. User Variability Assessment

To evaluate the impact of user experience on the iSperm system’s performance, semen samples with a concentration of approximately 43 million sperm/mL were selected. Three groups were tested: trained users operating iSperm, untrained users operating iSperm, and untrained users loading CASA slides (analyzed by a trained technician). Each user in the iSperm groups performed 20 replicate measurements, while the CASA group followed the same protocol for slide preparation. Box plot analysis was used to visualize the distribution and variability of measurements across groups, providing a comparative overview of user performance. Variability and user influence were further assessed by calculating the standard deviation (SD) and coefficient of variation (CV) across repeated measurements for each group.

#### 2.6.3. Analysis Procedure

For the Hemocytometer analysis, a 10 µL aliquot of diluted semen was loaded into a chamber with a depth of 100 µm (Neubauer double counting chamber, distributed by Gizmo Supply Co., Fort Lauderdale, USA; design originated Germany), and the evaluation was performed according to standard procedures as outlined in the provided guidelines. The iSperm system analysis followed the instructions, with adaptations made for boar semen. Approximately 50 µL of diluted semen was introduced into the iSperm cover chip, followed by enclosing it with the iSperm base chip. The assembled unit was then inserted into the lens module, which includes a heating chamber. Four different fields were captured and analyzed after rotating the chip set (0°, 90°, 180°, and 270°). For CASA analysis (HTM-CEROS II, Hamilton-Thorne Research, Beverly, MA, USA), a 3-µL aliquot of semen was loaded into a 19.7 µm Leja slide (Leja Products B.V., Nieuw-Vennep, The Netherlands) using capillary action. The slide was placed on a heated stage (37 °C) during analysis. Eight microscopic fields were selected to determine sperm concentration, while sixteen fields were analyzed to assess sperm motility and progressive motility. A detailed comparison of the three methods (iSperm, hemocytometer, and CASA) is provided in Table S1.

### 2.7. CASA Comparisons to Validate the iSperm Analyzer

Semen samples from 77 commercially available boar semen doses were transported under refrigeration at 16 °C and evaluated using the iSperm system at a testing center located within a commercial boar artificial insemination (AI) station. The AI station itself is part of a livestock production facility. The samples were pre-warmed to 37 °C by placing them in a water bath for 5 min before analysis. Sperm concentration, motility, and progressive motility were evaluated using both the traditional CASA system and the iSperm analyzer, with each analysis performed once per sample for comparison.

To evaluate the agreement between the iSperm analyzer and the CASA system, Passing–Bablok regression analysis and Bland–Altman plot were employed to assess the linear relationship and to determine the level of agreement between the two methods, respectively. The coefficient of variation (CV) was also calculated to measure the precision of the results. These statistical analyses were performed using MedCalc version 20.014.

An ROC analysis was conducted to assess the diagnostic performance of the iSperm analyzer in classifying semen samples based on farm-defined thresholds for sperm concentration, total motility, and progressive motility, using the CASA system as the reference standard. Sensitivity and specificity were calculated, and the area under the curve (AUC) was determined to quantify the analyzer’s ability to accurately distinguish samples. This ROC analysis, along with other statistical tests, was also performed using MedCalc version 20.014.

## 3. Results and Discussion

### 3.1. System Design and Characterization

The iSperm system offers notable advancements over traditional semen analysis methods through the integration of a precision-engineered optical module, a droplet-loaded microchip, a heater module, and an intuitive software interface. The optical module, optimized for compatibility with the iPad Mini 6, captures clear and detailed images necessary for accurate measurements of sperm concentration, motility, and progressive motility. The droplet-loaded microchip eliminates the need for manual pipetting by employing a specialized design that facilitates consistent sample loading. This design ensures a standardized sample thickness and observation area, minimizing the Segre-Silberberg (SS) effect and reducing operator-dependent errors, ultimately enhancing reproducibility compared to conventional counting chambers. The heater module sustains physiological temperature (~37.5 °C), a critical factor for reliable motility assessments, especially under field conditions. Combined with a software interface capable of analyzing sperm videos at 60 fps, the iSperm system mitigates the variability commonly associated with traditional CASA systems and delivers reproducible results suited for on-site applications. The system’s straightforward operation and reduced reliance on user expertise further support its practicality and effectiveness for field-based semen quality assessments.

### 3.2. Precision and User Variability Testing

**Figure 7:**
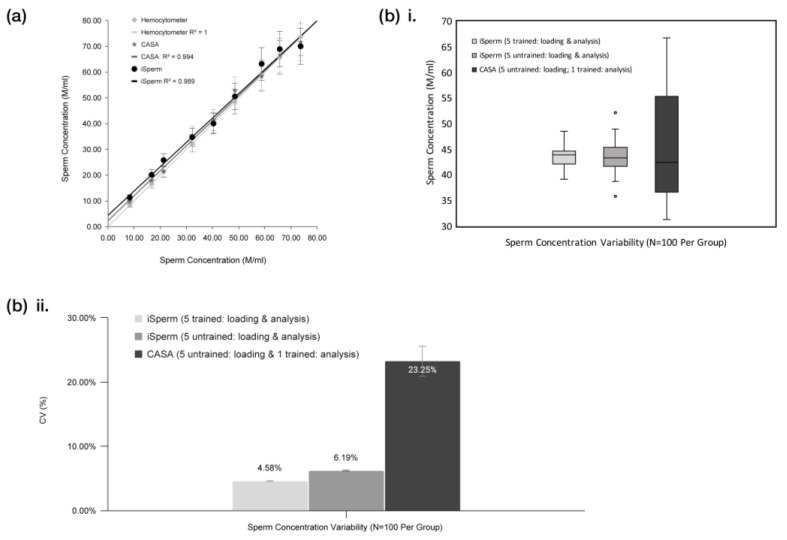
Precision and user variability testing. (**a**) Sperm concentration (M/mL) results obtained from the hemocytometer, iSperm, and CASA systems, for concentrations ranging from 10 to 75 M/mL. (**bi**) Box plot comparing median sperm concentration measurements by trained and untrained users using the iSperm analyzer, and by CASA with slides loaded by untrained users and analyzed by trained personnel. Trained iSperm users reported a median value of 43.91 M/mL (IQR: 2.59), untrained iSperm users had 43.28 M/mL (IQR: 3.74), and CASA analysis based on untrained user-loaded slides yielded 42.41 M/mL (IQR: 36.69). (**bii**) Coefficient of variation (CV) in sperm concentration measurements by trained users using iSperm (4.58%), untrained users using iSperm (6.19%), and untrained users loading CASA slides (23.25%, analysis performed by trained personnel), illustrating the variability observed across trained iSperm users, untrained iSperm users, and untrained CASA loading.

To evaluate the precision of the iSperm analyzer, we compared its sperm concentration measurements with traditional methods, including the hemocytometer and CASA systems, as well as assessed the impact of user experience on measurement variability. As shown in Figure 7a, sperm concentration results from the iSperm analyzer closely aligned with those obtained from the hemocytometer and CASA across a range of 10 M/mL to 75 M/mL. At lower concentrations (e.g., <20 M/mL), iSperm measurements showed minor divergence from hemocytometer and CASA values, with deviations up to approximately 2–3 M/mL. Nonetheless, the overall agreement remained strong across the full range of 10–75 M/mL, as indicated by R^2^ = 0.989, highlighting the iSperm analyzer’s ability to provide reliable concentration measurements comparable to established techniques.

To further evaluate the system’s robustness and user-dependent variability, five trained and five untrained users participated in a sperm concentration measurement test. Each user performed 20 replicate measurements using the iSperm analyzer, yielding 100 data points per group. In addition, the same five untrained users also performed slide loading for CASA analysis, with subsequent image acquisition and processing conducted by trained personnel to isolate the effect of sample preparation. As shown in Figure 7(bi), trained iSperm users reported a median sperm concentration of 43.91 M/mL (IQR: 2.59), while untrained iSperm users achieved a comparable median of 43.28 M/mL (IQR: 3.74). A third group—CASA slides prepared by untrained users—showed a median of 42.41 M/mL with a wider IQR of 36.69, suggesting greater variability introduced during manual slide loading. The coefficient of variation (CV) analysis (Figure 7b(ii)) confirmed these findings: trained and untrained iSperm users had CVs of 4.58% and 6.19%, respectively, whereas CASA measurements with untrained loading showed a higher CV of 23.25%. These results underscore the iSperm system’s ability to minimize user-induced variability through its droplet-loaded chip and streamlined workflow.

Overall, the results from Figure 7a,b confirm that the iSperm analyzer delivers consistent and reliable sperm concentration measurements, aligning well with conventional methods such as the hemocytometer and CASA systems. Furthermore, its precision is maintained across varying levels of user experience, reinforcing its suitability for routine use in both laboratory and field settings, where technical expertise may be limited.

### 3.3. Comparative Analysis Between iSperm and CASA

The comparison between the iSperm analyzer and the CASA system for both sperm concentration, motility and progressive motility was conducted using Passing–Bablok regression and Bland–Altman analysis.

#### 3.3.1. Passing–Bablok Regression Analysis

**Figure 8:**
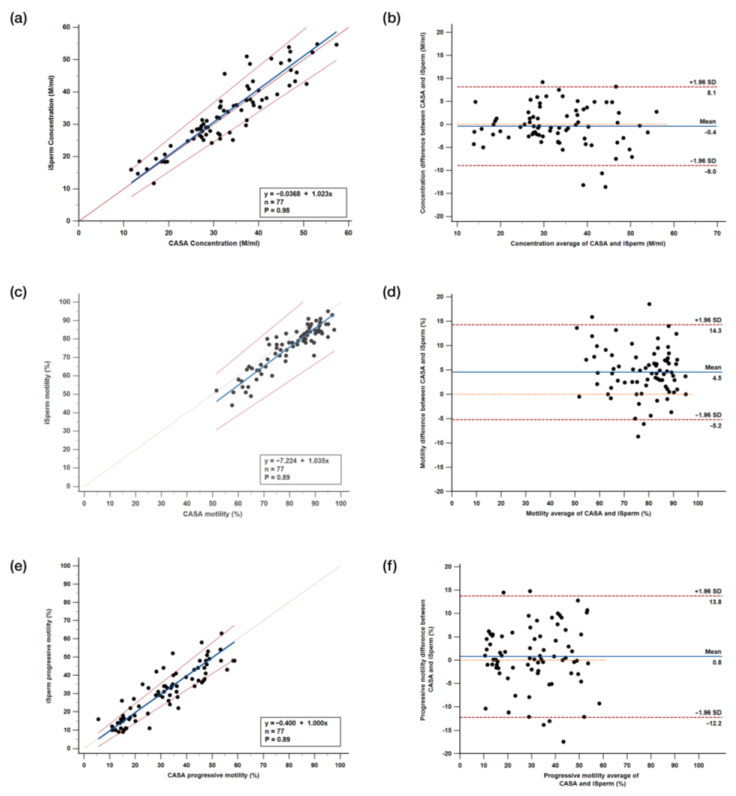
Comparative Analysis Between iSperm and CASA. Passing–Bablok analysis comparing (**a**) sperm concentration, (**c**) total motility, and (**e**) progressive motility as measured by iSperm and CASA across all boar semen samples. The solid blue line represents the regression line, the solid red line indicates the identity line, and the dashed red lines represent the confidence band. Bland–Altman analysis comparing (**b**) sperm concentration, (**d**) total motility, and (**f**) progressive motility between iSperm and CASA. In the Bland–Altman plots, the blue dotted lines represent the mean difference between the methods, while the red dashed line represents the 95% limits of agreement (LOAs).

Passing–Bablok regression analysis (n = 77) was conducted to evaluate the performance of the iSperm analyzer compared to CASA across all measured parameters. For sperm concentration (Figure 8a), the intercept (A) was −0.0368 (CI, −3.220 to 2.873) and the slope (B) was 1.023 (CI, 0.926 to 1.127). The CUSUM test for linearity indicated no significant deviation from linearity (*p* = 0.98), suggesting that iSperm closely aligns with CASA for concentration measurements. For total motility (Figure 8c), the intercept and slope values were −7.224 (CI, −17.039 to 1.707) and 1.035 (CI, 0.929 to 1.154), respectively, with no significant deviation from linearity (*p* = 0.89). Although the slope is slightly greater than 1, the negative intercept indicates that iSperm tends to slightly underestimate motility compared to CASA. In terms of progressive motility (Figure 8e), the intercept and slope were −0.400 (CI, −4.000 to 2.244) and 1.000 (CI, 0.897 to 1.111), with no significant deviation from linearity (*p* = 0.89). Given that progressive motility rarely exceeds 60% in the data, especially in regions like Southeast Asia where hot weather can negatively impact boar semen quality [5].

#### 3.3.2. Bland–Altman Analysis

The Bland–Altman plots further validate the agreement between iSperm and CASA, assessing bias and variability. For sperm concentration (Figure 8b), the mean difference is −0.4 M/mL, with most data points lying within the limits of agreement (+1.96 SD at 8.1 M/mL and −1.96 SD at −9.0 M/mL), indicating a minimal and acceptable systematic bias. For total motility (Figure 8d), the mean difference is 4.5%, with the limits of agreement set at +1.96 SD (14.3%) and −1.96 SD (−5.2%), indicating a slight negative bias by iSperm, though within clinically acceptable ranges. Finally, for progressive motility (Figure 8f), the mean difference is 0.8%, with the limits of agreement at +1.96 SD (13.8%) and −1.96 SD (−12.2%), signifying minimal bias and reinforcing the consistency between iSperm and CASA across different motility parameters.

CASA systems are widely regarded as the most accurate tools for semen analysis. However, due to variations in software and hardware across different models, there is no established standard, let alone a gold standard [12,13]. Table S1 provides a systematic approach for comparing the accuracy and correlation of various semen analysis methods with iSperm, ensuring that comparisons are conducted in a consistent and objective manner. Even among conventional CASA platforms, discrepancies in reported values are common. Sources of such variability include:

- Frame rate and number of analyzed frames;
- Geometry and optical depth of the specimen chamber;
- Algorithmic definitions and threshold settings (e.g., VAP, STR);
- Operator handling and environmental conditions (e.g., temperature).

In this study, we standardized key variables, including sample extender, chip height, frame rate, temperature, and operator training, to ensure a consistent comparison. However, the iSperm platform employs a custom image-processing algorithm that differs from those used in standard CASA systems. These algorithmic differences can inherently affect sperm trajectory detection and motion classification. Although we adopted the same motility thresholds used in the Hamilton Thorne CASA system (VAP ≥ 45 µm/s), the optical and analytical differences between platforms likely led to slight underestimation by iSperm. Applying uniform CASA criteria across systems without threshold calibration may cause sperm with borderline motility to be excluded in iSperm analysis.

To address this, future versions of the iSperm software will incorporate user-configurable threshold settings (e.g., VAP, STR), enabling better calibration across different systems and experimental conditions. This flexibility will help align iSperm outputs with those of various CASA platforms while accounting for differences in chip design, sample type, and analytical environment.

In conclusion, the strong correlations from the Passing–Bablok analysis and the minimal biases observed in the Bland–Altman plots confirm that the iSperm analyzer provides reliable and consistent measurements for sperm concentration, total motility, and progressive motility. These findings demonstrate iSperm’s suitability for on-site semen analysis, particularly in field settings where accurate assessments are crucial. The system’s ability to produce comparable results with CASA makes it an effective tool for real-time semen analysis in practical breeding environments.

### 3.4. Effect Evaluation

To further validate the iSperm system’s diagnostic capabilities, the system’s ability to distinguish between positive and negative motility outcomes was evaluated through scatter plot analysis and ROC curve analysis.

#### 3.4.1. Scatter Plot Analysis

**Figure 9:**
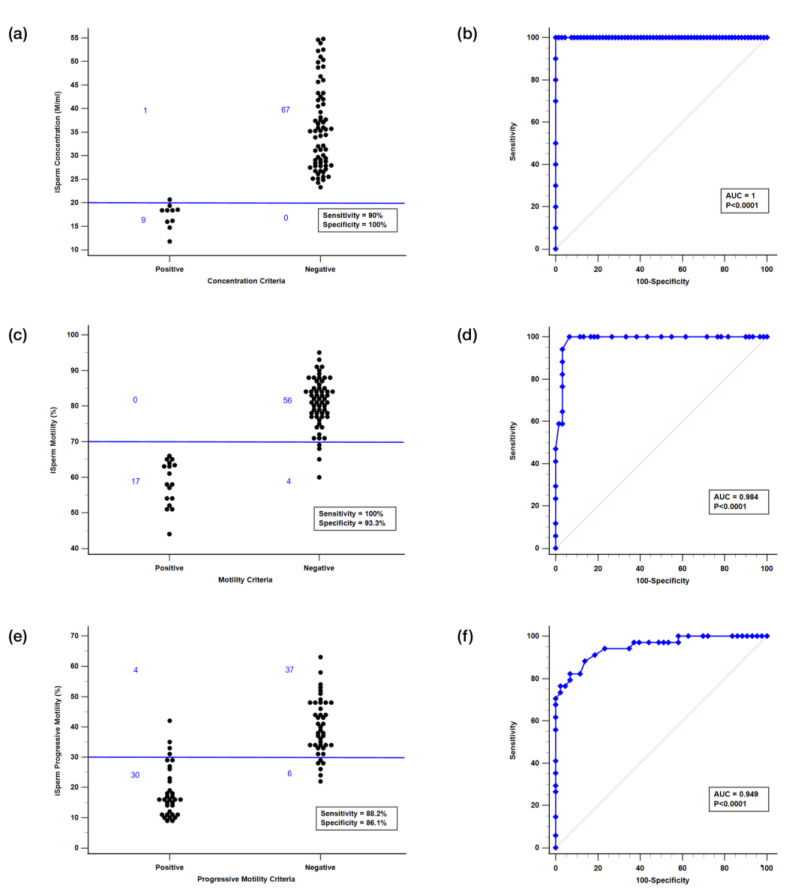
Effect evaluation. Device evaluation using boar semen samples based on farm-defined thresholds: 30 M/mL for concentration, 70% for total motility, and 30% for progressive motility. (**a**,**c**,**e**) Scatter plots of sperm concentration, total motility, and progressive motility, respectively, showing “Positive” and “Negative” groups separated by the blue threshold line. (**b**,**d**,**f**) Corresponding ROC curves illustrating the diagnostic performance of iSperm for each parameter. Area under the curve (AUC) values confirm the system’s high classification accuracy.

As shown in Figure 9a,c,e, scatter plots illustrate the distribution of iSperm measurements for sperm concentration, motility, and progressive motility across binary outcome groups (positive vs. negative), based on farm-defined thresholds: 30 M/mL for concentration, 70% for motility, and 30% for progressive motility. Negative samples predominantly cluster below these thresholds, while positive samples fall above, indicating clear separation. At these cutoffs, iSperm achieved sensitivity/specificity values of 90%/100% for concentration, 100%/93.33% for motility, and 88.2%/86.1% for progressive motility. These results demonstrate reliable classification performance, as supported by the clustering of values around the diagnostic thresholds, and support the applicability of iSperm in routine semen evaluation scenarios. As discussed in previous reviews of CASA systems [12,13], inter-system discrepancies are common due to differences in hardware components, software algorithms, optical configurations, and motion thresholds. Although we applied the same motility criteria as the Hamilton Thorne CASA system (VAP ≥ 45 µm/s and STR ≥ 45%), differences in iSperm’s image-processing algorithm likely contributed to deviations in progressive motility assessment. Applying uniform thresholds across heterogeneous platforms can result in systematic misclassification, particularly for borderline cases.

To address this limitation, we have included the following clarifications and proposed future developments:

- The current iSperm algorithm employs fixed thresholds for binary classification. Minor deviations in borderline cases can lead to misclassification. To improve flexibility, we plan to implement adjustable cutoff options (e.g., user-defined thresholds for progressive motility) to accommodate field-specific requirements.
- Future versions will also incorporate a confidence interval-based “gray zone” near classification thresholds to reduce volatility in borderline classification and enhance diagnostic robustness.

#### 3.4.2. ROC Curve Analysis

ROC curves (Figure 9b,d,f) further validated the system’s diagnostic accuracy across the same parameters. The area under the curve (AUC) exceeded 0.94 for all three: 1.000 (95% CI: 0.953–1.000) for concentration and 0.984 (95% CI: 0.925–0.999) motility, and 0.949 (95% CI: 0.874–0.986) for progressive motility. The corresponding classification accuracies were 98.7% for concentration, 94.8% for motility, and 87.1% for progressive motility. The consistently high AUC values (>0.95) across all parameters indicate that iSperm can effectively distinguish between acceptable and substandard samples. These findings support its utility as a practical tool for routine semen quality assessment, particularly in field-based swine artificial insemination programs.

## 4. Conclusions

The iSperm system provides a reliable and accessible solution for boar semen quality assessment before AI, offering portability and ease of use comparable to standard laboratory equipment. The innovative droplet-loaded microchip streamlines sample preparation, reducing user error with an intuitive design that captures precise semen volumes without the need for additional pipetting. The combination of the optical module, integrated heater, and guided software interface enhances the system’s performance, enabling consistent and accurate measurements of sperm concentration, motility and progressive motility.

Comparative analyses between iSperm and CASA systems, using Passing–Bablok regression and Bland–Altman plots, demonstrated strong agreement between the methods, with minimal bias and acceptable variability limits. Notably, the system’s performance remained robust across both trained and untrained users, underscoring its potential for broad applications. ROC curve analysis confirmed the system’s high diagnostic accuracy across multiple semen quality parameters. Its diagnostic capabilities position iSperm as a valuable tool for reproductive health monitoring and animal breeding programs, paving the way for broader adoption across diverse settings. Beyond boar semen, iSperm has also been validated in other species, such as stallions, dogs, and rhinoceroses. Previous studies have shown the effectiveness of various generations of the iSperm system in accurately assessing sperm concentration, motility, and progressive motility in equine, canine, and exotic species [34,35,36,37,38,39,40,41,42,43,44].

Despite this, iSperm’s versatility as a reliable tool for reproductive monitoring across species facilitates routine semen evaluation in both laboratory and field environments. This paper presents a new generation of the iSperm system, tailored to a novel application, demonstrating its ongoing evolution and adaptability to meet emerging needs in reproductive analysis. Looking ahead, the platform could potentially be adapted for broader applications, such as male infertility diagnostics, cell counting in medical settings, and pathogen detection in environmental monitoring, expanding its utility beyond reproductive analysis.

## 5. Patents

The authors declare that a patent application related to the iSperm system described in this study has been filed. This does not affect the objectivity or integrity of the presented research. Parts of the technology are protected by issued patents in multiple jurisdictions: Taiwan Patent No. 102146719, U.S. Patent No. 14/921,494., Japan Patent No. 2015-548049, Korea Patent No. 10-2015-7018113, and China Patent No. 201380065817.0.

## Data Availability

Data are all contained within the article.

## Supplementary Materials

The following supporting information can be downloaded at: https://www.mdpi.com/article/10.3390/bios15060394/s1. Figure S1. Illumination Module Design and Components. Figure S2. Sperm images from different species captured using the iSperm system. Figure S3. Exploded assembly view of the iSperm hardware system. Table S1. Evaluation Methods and Process Flow for Sperm Analysis Using Different Systems. Table S2. Sperm Concentration (M/mL) Measured Using Hemocytometer, iSperm, and CASA Across a Range of 10–75 M/mL. Table S3. Repeated Measurements on the Same Sample by Trained Users operating iSperm and Untrained Users operating iSperm. Table S4. Repeated Measurements on the Same Sample by Untrained Users loading CASA slides (analyzed by a trained technician). Table S5. Sperm Concentration, Motility, and Progressive Motility Measured by iSperm and CASA. Table S6. Technical and Functional Comparison of iSperm with Existing Semen Analysis Platforms. Video S1 (.mp4 format). iSperm Semen Sampling Video. Video S2 (.mp4 format). iSperm Software Analysis Video.
