# Supplementary material for: A Mobile Sperm Analyzer with User-Friendly Microfluidic Chips for Rapid On-Farm Semen Evaluation
Source: Biosensors (Basel). 2025 Jun 18;15(6):394. doi: 10.3390/bios15060394 (PMC12190950; doi:10.3390/bios15060394)
Supplement: Supplementary file 1 [file biosensors-15-00394-s001.zip › biosensors-3662887-supplementary.pdf]

Supplementary Materials for

A Mobile Sperm Analyzer with User-Friendly Microfluidic Chips for Rapid On-Farm Semen Evaluation

Shu-Sheng Li[1,2], Chang-Yu Chen[2], Cheng-Ming Lin[2], Tsun-Chao Chiang[2], Yu-Siang Tang[2], Chang-Ching Yeh[2], Wei-Fan Hsu[1] and Andrew M. Wo[1]*

[1] *Institute of Applied Mechanics, National Taiwan University, Taipei, Taiwan, Republic of China*

[2] *Aidmics Biotechnology, Taipei, Taiwan, Republic of China*

The PDF file includes:
Figure S1. Illumination Module Design and Components.
Figure S2. Sperm images from different species captured using the iSperm system.
Figure S3. Exploded assembly view of the iSperm hardware system.

Table S1. Evaluation Methods and Process Flow for Sperm Analysis Using Different Systems.
Table S2. Sperm Concentration (M/mL) Measured Using Hemocytometer, iSperm, and CASA Across a Range of 10–75 M/mL.
Table S3. Repeated Measurements on the Same Sample by Trained Users operating iSperm and Untrained Users operating iSperm.
Table S4. Repeated Measurements on the Same Sample by Untrained Users loading CASA slides (analyzed by a trained technician).
Table S5. Sperm Concentration, Motility, and Progressive Motility Measured by iSperm and CASA.
Table S6. Technical and Functional Comparison of iSperm with Existing Semen Analysis Platforms

Other Supplementary Material for this manuscript includes the following: available at
https://doi.org/10.6084/m9.figshare.29314973 (accessed on 13 June 2025)

Video S1 (.mp4 format). iSperm Semen Sampling Video.
Video S2 (.mp4 format). iSperm Software Analysis Video.

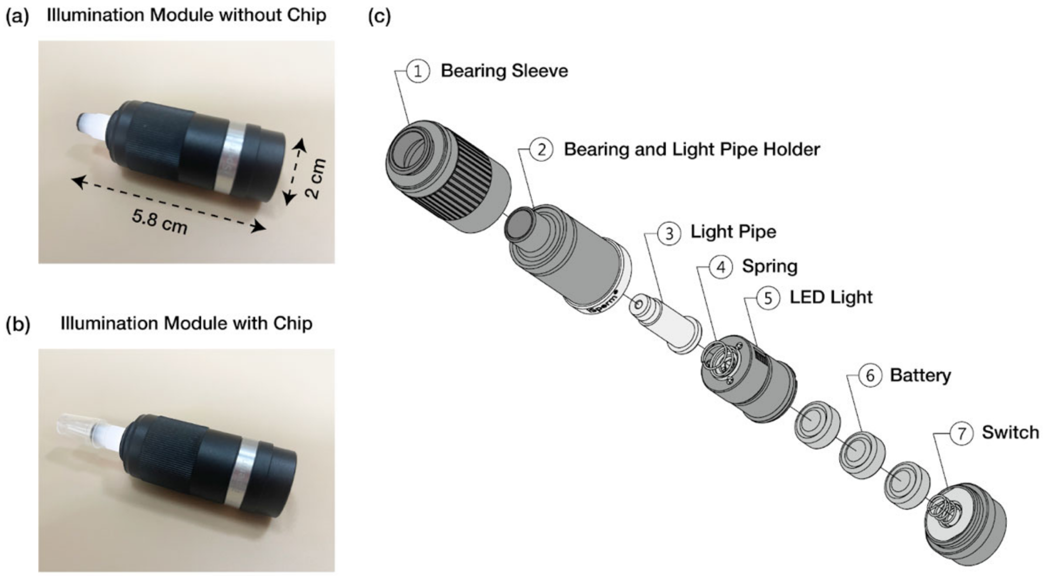

Figure S1. Illumination Module Design and Components. The module integrates an LED-based lighting system and a light pipe to provide consistent illumination for sperm sample imaging while holding the chip securely in place. (a) Illumination Module without Chip: The illumination module without the chip installed, measuring 5.8 cm × 2 cm. (b) Illumination Module with Chip: The illumination module with chip installed for sample analysis. (c) Exploded View of the Illumination Module.

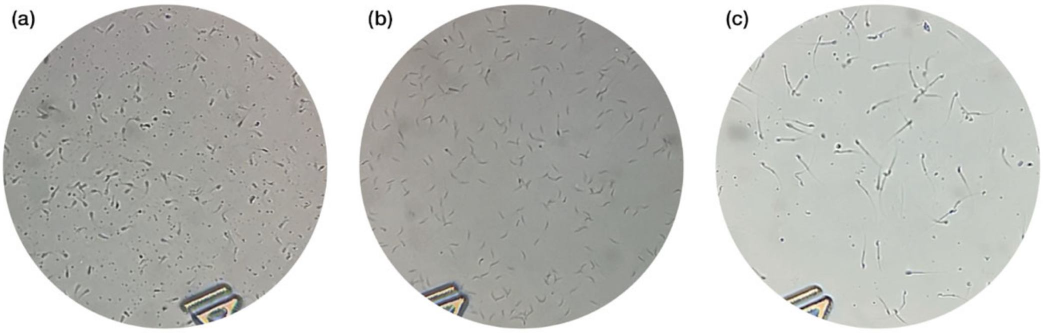

Figure S2. Sperm images from different species captured using the iSperm system. (a) Canine sperm: Dog sperm cells showing typical elongated heads and slender tails. (b) Avian sperm: Chicken sperm cells characterized by their thin, filament-like structures. (c) Murine sperm: Mouse sperm cells displaying distinctive hook-shaped heads and long tails.

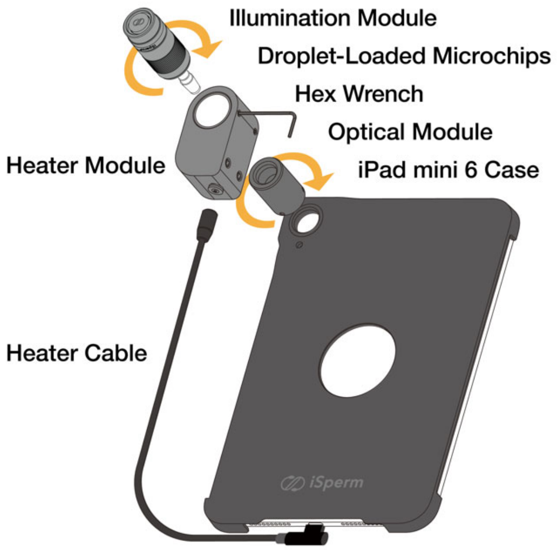

Figure S3. Exploded assembly view of the iSperm hardware system. The assembly process begins by attaching the Optical Module to the iPad mini 6 Case. The Heater Module is then mounted onto the Optical Module and secured in place using the Hex Wrench. The Heater Cable is subsequently connected to power the thermal component. After sample preparation, the Droplet-Loaded Microchip, together with the Illumination Module (guide light rod), is positioned and fastened onto the top of the Optical Module, completing the alignment along the optical axis.

Video S1. Video demonstrating the semen sampling procedure using the iSperm system. The video illustrates step-by-step the collection, preparation, and loading of boar semen samples onto the disposable droplet-loaded microchip.

Video S2. Video demonstrating the software analysis process using the iSperm system. The video showcases the user interface of the iSperm application, detailing how to initiate and monitor the analysis. It includes the steps for selecting the analysis mode, inputting sample information, and viewing the real-time assessment of boar semen concentration, motility and progressive motility.

Table S1. Evaluation Methods and Process Flow for Sperm Analysis Using Different Systems.

Equipment	Counting Chamber & Sampling Volume	Evaluation Range	Evaluation Method	Motility Readings & Progressive Motility Readings
Phase Contrast Microscope	Hemocytometer<br>For Concentration<br>- Volume: 10 uL x 2 repeats<br>- Depth: 100um<br><br>Glass Slide<br>For Motility<br>- Volume: 10uL	0 - 10 M/ml	Hemocytometer<br>Perform 2 repetitions of the test, with each repetition evaluating<br>both sides of the chamber.<br>Each evaluation involves examining 5 large squares, each of which contains 16 smaller squares.<br><br>**The minimum sperm count for each side should be over 200 million, the test should be repeated or resampled**<br><br>Glass Slide<br>Please refer to WHO guidelines.	Motility:<br>Massal: Category from 1 - 5<br>Individual: Percentage %<br><br>Progressive Motility:<br>Evaluation is not feasible due to limitations of subjective observations.
CASA System	Leja Counting Chamber<br>For Concentration & Motility & Progressive Motility<br>- Volume: 3uL<br>- Depth: 20um (recommended)	20 – 60 M/ml	Perform 3 repetitions, evaluate a total of 8 views of the Leja counting chamber, which are arranged in 2 columns and 4 rows, and use their average for Concentration.<br>Perform 3 repetitions, evaluate a total of 16 views of the Leja counting chamber, which are arranged in 2 columns and 8 rows, and use their average for motility and Progressive motility.	Motility:<br>Percentage %, using a set of distinct Kinetics Parameters depending on the Hamilton Thorne CASA system (Motility：VAP≥45).<br><br>Progressive Motility:<br>Percentage % & Sperm Tracks, based on a set of distinct Kinetics Parameters depending on the Hamilton Thorn CASA system.
iSperm System	iSperm Base & Cover Chips<br>For Concentration & Motility & Progressive Motility<br>- Volume: A Drop (~50uL)<br>- Depth: 20um	10 – 75 M/ml (For Sperm Tracking)	Perform 3 repetitions of the evaluation, using different chips for each test and capturing 4 fields of view for each repetition, and use their average.<br>** Complete evaluation within 1 min after chip sampling**	Motility:<br>Percentage % and visualized sperm tracks based on defined kinetic parameters. Parameters depending on the Hamilton Thorn CASA system. (Motility：VAP≥45).<br><br>Progressive Motility:<br>Percentage % and visualized sperm tracks based on defined kinetic parameters, with default kinetics parameters settings based on Hamilton Thorne CASA system. (Progressive Motility：VAP≥45, STR≥45).

Table S2. Sperm Concentration (M/mL) Measured Using Hemocytometer, iSperm, and CASA Across a Range of 10–75 M/mL.

Sample	Repeat Measurement	Hemocytometer (M/ml)	iSperm (M/ml)	CASA (M/ml)
1	Measurement 1	8.02	10.00	14.44
	Measurement 2	8.66	11.00	13.37
	Measurement 3	NA	8.00	6.15
2	Measurement 1	16.35	17.00	19.62
	Measurement 2	17	18.00	19.82
	Measurement 3	NA	19.00	21.00
3	Measurement 1	20.88	22.47	24.93
	Measurement 2	21.70	21.11	25.74
	Measurement 3	NA	20.88	26.77
4	Measurement 1	31.25	34.12	38.95
	Measurement 2	33.25	38.70	35.02
	Measurement 3	NA	33.65	30.28
5	Measurement 1	40.18	40.67	41.25
	Measurement 2	40.56	41.71	39.89
	Measurement 3	NA	41.67	38.98
6	Measurement 1	49.79	53.15	55.48
	Measurement 2	47.32	53.41	49.41
	Measurement 3	NA	52.09	46.77
7	Measurement 1	59.30	60.20	61.88
	Measurement 2	58.27	56.20	64.78
	Measurement 3	NA	58.70	62.90
8	Measurement 1	65.43	67.94	68.38

	Measurement 2	66.00	65.48	70.67
	Measurement 3	NA	66.22	67.74
9	Measurement 1	74.45	72.00	70.22
	Measurement 2	72.95	71.00	74.65
	Measurement 3	NA	72.00	65.21

Table S3. Repeated Measurements on the Same Sample by Trained Users operating iSperm and Untrained Users operating iSperm.

Repeat Measurement	Trained Users 1	Trained Users 2	Trained Users 3	Trained Users 4	Trained Users 5	Untrained Users 1	Untrained Users 2	Untrained Users 3	Untrained Users 4	Untrained Users 5
1	42.40	45.66	47.10	44.10	46.24	41.70	39.18	41.69	43.5	45.69
2	45.30	46.90	46.10	46.50	40.94	44.80	41.83	41.37	39.5	47.18
3	43.90	43.80	43.10	45.00	44.42	45.00	45.98	44.71	45.1	44.12
4	42.90	44.40	44.00	42.60	44.10	43.00	46.49	44.06	38.7	45.55
5	41.90	40.80	44.90	44.30	40.48	43.00	44.91	41.73	41.6	42.51
6	46.00	44.70	44.10	39.96	44.19	47.30	46.36	40.85	43.0	43.56
7	40.30	43.00	46.80	44.10	44.10	38.70	43.98	42.22	46.3	43.45
8	45.90	42.90	40.90	42.10	43.65	45.90	47.69	42.65	42.5	42.21
9	43.30	43.80	45.37	44.60	41.77	43.40	47.60	35.81	41.4	39.57
10	43.00	40.60	43.60	43.60	45.52	46.50	40.80	41.76	45.0	40.20
11	44.70	44.50	42.49	41.80	41.81	42.40	45.80	40.73	43.37	42.95
12	44.10	45.20	46.58	45.60	39.96	39.70	39.83	40.28	40.90	43.59
13	42.80	48.50	44.19	42.20	42.13	40.00	48.26	52.20	41.10	39.33
14	44.40	44.00	44.57	43.70	43.94	42.10	41.09	42.30	44.00	44.69
15	41.95	43.90	44.30	42.50	39.39	41.40	45.62	44.08	44.00	43.04
16	43.70	42.80	45.00	40.40	39.57	41.70	46.58	41.58	44.20	45.56
17	44.00	43.90	44.60	46.80	41.78	41.70	40.48	40.11	45.20	48.10

18	46.40	43.10	39.40	45.60	39.17	47.60	45.94	43.19	44.90	48.10
19	40.70	43.40	44.70	42.00	44.30	42.50	42.73	45.42	45.60	49.00
20	41.50	46.40	45.40	41.10	40.97	44.00	43.47	42.65	44.80	43.11

Table S4. Repeated Measurements on the Same Sample by Untrained Users loading CASA slides (analyzed by a trained technician).

Repeat Measurement	Untrained Users 1	Untrained Users 2	Untrained Users 3	Untrained Users 4	Untrained Users 5
1	55.00	62.24	32.68	39.7	57.9
2	43.47	51.63	62.41	44.3	34.7
3	42.43	62.06	45.61	35.6	39.0
4	42.37	43.19	66.79	36.6	57.7
5	65.93	35.13	55.37	34.2	48.2
6	35.73	62.35	31.90	38.4	36.9
7	44.72	64.17	44.22	31.4	53.5
8	44.32	43.19	31.38	38.6	40.5
9	36.13	66.79	62.54	41.8	41.0
10	33.70	55.37	34.17	34.2	37.3
11	42.47	54.22	50.00	50.0	44.1
12	42.37	41.70	60.00	39.9	37.5
13	43.91	36.30	36.57	36.6	59.4
14	64.61	55.58	35.73	35.7	31.3
15	32.06	43.67	33.00	56.1	56.9
16	63.00	37.17	39.60	39.6	38.8
17	66.83	43.26	42.47	42.5	42.4
18	55.82	66.49	38.09	58.1	33.7
19	45.13	37.78	63.30	43.3	35.5
20	34.80	41.89	41.05	41.1	37.8

Table S5. Sperm Concentration, Motility, and Progressive Motility Measured by iSperm and CASA.

Sample	Concentration (M cells/ml)		Motility (%)		Progressive Motility (%)	
	iSperm	CASA	iSperm	CASA	iSperm	CASA
1	35.73	34.46	79.00	83.30	33.00	32.50
2	35.20	31.40	74.00	76.50	23.00	21.40
3	30.06	27.72	71.00	78.60	19.00	24.90
4	29.40	27.26	60.00	73.20	16.00	15.70
5	18.40	19.69	61.00	69.00	17.00	15.30
6	18.40	18.40	49.00	64.90	16.00	14.40
7	43.27	38.75	93.00	96.70	49.00	48.90
8	37.67	38.11	90.00	90.40	28.00	30.60
9	37.40	37.30	91.00	87.30	31.00	29.00
10	37.10	31.60	89.00	91.90	48.00	45.20
11	25.11	27.72	79.00	88.50	34.00	34.20
12	11.77	16.68	84.00	86.90	33.00	33.90
13	41.78	37.47	91.00	92.00	53.00	48.40
14	32.11	30.72	87.00	88.10	41.00	35.90
15	51.00	37.38	95.00	95.00	63.00	53.70
16	27.82	27.44	84.00	91.40	48.00	53.50
17	50.33	42.85	87.00	91.50	34.00	31.70
18	35.23	31.36	88.00	94.20	58.00	45.90
19	27.83	27.44	83.00	90.00	34.00	36.20
20	28.48	26.70	84.00	90.80	29.00	28.00
21	40.50	40.00	88.00	87.00	44.00	31.00
22	29.13	28.70	86.00	87.60	36.00	46.00
23	18.37	19.15	84.00	92.40	48.00	47.60
24	20.68	19.15	82.00	85.20	40.00	34.80
25	24.83	24.25	81.00	85.30	37.00	46.60
26	23.27	20.42	79.00	85.30	29.00	33.50

27	26.07	27.35	85.00	91.30	43.00	43.00
28	27.80	25.16	83.00	85.90	34.00	31.20
29	26.77	31.36	83.00	92.70	43.00	55.80
30	28.39	26.26	81.00	84.40	31.00	36.10
31	27.16	31.00	85.00	89.70	38.00	47.10
32	26.50	26.99	78.00	89.50	29.00	29.40
33	29.70	37.20	88.00	95.10	48.00	58.70
34	27.90	29.20	82.00	86.90	35.00	34.60
35	29.02	28.26	84.00	88.10	44.00	44.50
36	31.29	27.44	84.00	82.70	31.00	31.20
37	31.05	28.45	88.00	88.50	52.00	34.60
38	16.12	15.13	57.00	61.40	22.00	18.10
39	14.73	13.13	54.00	63.90	16.00	5.60
40	18.50	13.49	68.00	78.40	11.00	16.20
41	15.98	11.67	77.00	75.00	14.00	14.10
42	48.89	44.95	66.00	68.80	16.00	17.60
43	54.60	57.35	58.00	60.10	9.00	13.50
44	54.80	53.06	77.00	72.00	15.00	15.10
45	52.24	51.97	65.00	64.20	11.00	16.50
46	52.49	47.04	63.40	63.40	12.00	11.00
47	43.31	48.14	51.00	58.10	9.00	15.20
48	46.80	47.14	52.00	51.50	10.00	11.00
49	42.48	50.69	81.00	95.00	37.00	44.70
50	36.74	38.75	82.00	85.20	51.00	48.00
51	31.27	37.38	71.00	89.50	38.00	42.20
52	40.92	37.84	80.00	71.30	42.00	28.20
53	35.26	40.30	88.00	91.80	54.00	53.30
54	39.24	44.13	85.00	97.40	48.00	58.20
55	19.36	17.14	78.00	86.30	44.00	46.90
56	35.59	31.55	77.00	77.10	41.00	47.50

57	31.95	31.27	76.00	81.40	46.00	47.90
58	34.18	32.27	80.00	85.90	18.00	19.80
59	35.90	38.93	72.00	74.50	17.00	18.00
60	38.10	41.76	65.00	70.70	14.00	13.10
61	25.53	25.53	75.00	75.00	35.00	22.90
62	53.89	46.77	64.00	65.30	16.00	15.00
63	46.01	48.50	63.00	68.20	15.00	20.09
64	25.50	31.45	51.00	62.90	11.00	25.50
65	37.52	40.75	63.00	67.30	18.00	15.20
66	49.82	46.86	54.00	61.70	10.00	12.30
67	42.01	46.86	58.00	67.10	10.00	15.60
68	28.79	27.08	44.00	57.60	11.00	14.40
69	26.75	28.35	78.00	79.80	33.00	25.10
70	34.41	36.19	83.00	78.60	27.00	19.40
71	35.99	35.10	69.00	71.40	26.00	14.80
72	33.90	33.16	80.00	86.20	28.00	36.50
73	27.46	33.59	77.00	83.00	39.00	39.80
74	24.24	29.58	74.00	78.90	34.00	43.10
75	45.65	32.45	81.00	74.90	24.00	33.50
76	48.70	38.05	78.00	81.00	22.00	36.80
77	25.11	34.29	71.00	76.10	26.00	33.00

Table S6. Technical and Functional Comparison of iSperm with Existing Semen Analysis Platforms

**Category/Source**<br>**Item/Feature**		**Lin et al. (N/A)**	**Zheng et al. (2023) [22]**	**Trujillo et al. (2022) [23]**	**Kanakasabapathy et al. (2017) [24]**	**Agarwal et al. (2018) [27]**	**Hamilton Thorne**	**Medical Electronic Systems**
**Devices (Technology)**		iSperm (Smartphone +Microfluidic)	Smartphone +Microfluidic	Fertile-Eyez (Smartphone +Microfluidic)	Smartphone +Microfluidic	Yo Sperm (Smartphone +Microfluidic)	CASA (Microscope +Microfluidic)	SQA (Electro optics +Microfluidic)
**1. Droplet-Loaded**		V	X (Capillary Loading)	X (Capillary Loading)	X (Capillary Loading)	X (Capillary Loading)	X (Capillary Loading)	X (Capillary Loading)
**2. Built-in heater**		V	X	X	X	X	V	V
**4. Portable**		V	V	V	V	V	X	X
**5. User Variability**		V	N/A	N/A	V	V	X	X
**6. Rapid (<1min)**		V	N/A	N/A	V	V	X	V
**7. Species**		Boar	Boar	Boar	Human	Human	Boar<br>Human	Boar<br>Human
**8. Usage Environment**		Farm	Farm	Farm	Home use	Home use	Laboratory	Laboratory
**9. Parameters**	**Concentration**	V	X	V	V	V	V	V
	**Motility**	V	V	X (r = 0.558)	V	V	V	V
	**Progressive Motility**	V	V	X	X	X	V	V
	**Morphology**	X	X	V	X	X	V	X
**10. Price (USD)**		<$3,500	N/A	N/A	N/A	<$100 (Per test)	>$100,000	>$10,000

*** **V** = Feature supported or implemented; **X** = Feature not supported or absent; **N/A** = Not reported or insufficient information available. *** Blue = iSperm (this study); Green = smartphone-based platforms in literature; Pink = commercial CASA systems.
